# Transmission characteristics of heterozygous cases of Creutzfeldt-Jakob disease with variable abnormal prion protein allotypes

**DOI:** 10.1186/s40478-020-00958-x

**Published:** 2020-06-09

**Authors:** Anne Ward, Jason R. Hollister, Kristin McNally, Diane L. Ritchie, Gianluigi Zanusso, Suzette A. Priola

**Affiliations:** 1 Rocky Mountain Laboratories, Laboratory of Persistent Viral Diseases, National Institute of Allergy & Infectious Diseases, National Institutes of Health, 903 S. 4th Str, Hamilton, MT 59840 USA; 2 Rocky Mountain Laboratories, Laboratory of Virology, National Institute of Allergy & Infectious Diseases, National Institutes of Health, Hamilton, MT USA; 3 National CJD Research & Surveillance Unit, Centre for Clinical Brain Sciences, School of Clinical Sciences, University of Edinburgh, Edinburgh, UK; 4 Department of Neurosciences, Biomedicine, and Movement Sciences, University of Verona, Verona, Italy

**Keywords:** Prion, Creutzfeldt-Jakob disease, CJD, PrP^Sc^ allotype

## Abstract

In the human prion disease Creutzfeldt-Jakob disease (CJD), different CJD neuropathological subtypes are defined by the presence in normal prion protein (PrP^C^) of a methionine or valine at residue 129, by the molecular mass of the infectious prion protein PrP^Sc^, by the pattern of PrP^Sc^ deposition, and by the distribution of spongiform change in the brain. Heterozygous cases of CJD potentially add another layer of complexity to defining CJD subtypes since PrP^Sc^ can have either a methionine (PrP^Sc^-M129) or valine (PrP^Sc^-V129) at residue 129. We have recently demonstrated that the relative amount of PrP^Sc^-M129 versus PrP^Sc^-V129, i.e. the PrP^Sc^ allotype ratio, varies between heterozygous CJD cases. In order to determine if differences in PrP^Sc^ allotype correlated with different disease phenotypes, we have inoculated 10 cases of heterozygous CJD (7 sporadic and 3 iatrogenic) into two transgenic mouse lines overexpressing PrP^C^ with a methionine at codon 129. In one case, brain-region specific differences in PrP^Sc^ allotype appeared to correlate with differences in prion disease transmission and phenotype. In the other 9 cases inoculated, the presence of PrP^Sc^-V129 was associated with plaque formation but differences in PrP^Sc^ allotype did not consistently correlate with disease incubation time or neuropathology. Thus, while the PrP^Sc^ allotype ratio may contribute to diverse prion phenotypes within a single brain, it does not appear to be a primary determinative factor of disease phenotype.

## Introduction

Prion diseases are fatal, transmissible neurodegenerative diseases of mammals that are associated with the misfolding of a normally protease-sensitive and soluble protein called prion protein or PrP^C^, into a partially protease-resistant, insoluble and infectious form termed PrP^Sc^ [18]. Accumulation of PrP^Sc^ in the brain over time, as large amyloid or non-amyloid aggregates, eventually leads to clinical prion disease and death. In humans, prion diseases can be hereditary, sporadic, or acquired. In hereditary prion diseases, mutations in the prion protein (*PRNP*) gene are associated with different types of prion disease such as Gerstmann-Sträussler-Scheinker syndrome, fatal familial insomnia, and genetic Creutzfeldt-Jakob disease [26]. Sporadic Creutzfeldt-Jakob disease (sCJD), which is thought to arise as the result of spontaneous misfolding of PrP^C^ in the brain into infectious PrP^Sc^, is the most common form of human prion disease and occurs at an incidence of approximately1–2 cases per million people worldwide. Acquired forms of prion disease can result from the ingestion of prion contaminated tissue, as was the case with variant CJD which has been linked to ingestion of bovine spongiform encephalopathy (BSE) contaminated products, and kuru, which was the result of the cannibalistic practices of the Fore tribe in New Guinea. They can also result from exposure to prion contaminated medical instruments, devices or products such as dura mater grafts, pituitary gland derived human growth hormone, and human blood and blood products. Collectively, these latter forms of acquired prion disease are known as iatrogenic CJD (iCJD) [22].

In humans, there is a naturally occurring polymorphism in *PRNP* represented by either a methionine or valine at codon 129 which helps to define the neuropathological subtype of sCJD [25, 30, 31]. CJD subtypes are also classified according to the size of PrP^Sc^ following protease digestion [30–32, 41]. Type 1 CJD is associated with a PrP^Sc^ molecule of 21 kDa while Type 2 CJD is associated with a 19 kDa PrP^Sc^ molecule [30, 31]. There are therefore 6 potential subtypes of sCJD based on the possible combinations of PrP^Sc^ size and *PRNP* genotype: MM1, MM2, VV1, VV2, MV1, and MV2. CJD subtype can be further defined by different patterns of pathology (i.e. spongiform change and neuronal loss) and PrP^Sc^ deposition in the brain [3, 30, 31, 33], and by transmission properties into non-human primates [8] or transgenic mice expressing human PrP^C^ [1, 2, 5, 9, 13, 23, 44]. Based on all of these criteria, there are currently believed to be 6 major subtypes or strains of sCJD (in order of frequency): MM1/MV1, VV2, MV2K (i.e. Type 2 PrP^Sc^ with the “K” designating kuru-type plaques), MM2T (thalamic), MM2C (cortical), and VV1 [3].

Within CJD subtypes, there can be atypical disease presentations as well as variation in neuropathological phenotypes [3, 33, 34]. The molecular mechanisms underlying this variability are unclear but may be explained by the fact that multiple PrP^Sc^ types can co-occur within different CJD subtypes [10, 19, 24, 31, 34, 36, 41]. Approximately one-third of sCJD cases show the co-presence of Type 1 and Type 2 PrP^Sc^ within the same, or different, regions of a single brain [10, 34, 36]. For example, the most common mixed CJD type, MV1 + 2C, refers to cases with Type 1 PrP^Sc^ that also have cortical pathology (designated by the C) and focal Type 2 PrP^Sc^ deposition [33]. Thus, within a single subtype of sCJD, prions with different conformations may be present that could influence disease progression and pathogenesis in unpredictable ways.

The prion protein allotype may also be influencing disease phenotype. It is known that heterozygosity in PrP^C^ at codon 129 is associated with resistance to sCJD, as well as later disease onset and longer disease duration in kuru [11]. In sCJD, a valine at residue 129 has been associated with plaque formation and a longer clinical disease course [21] while a methionine at residue 129 has been associated with a more synaptic pattern of PrP^Sc^ deposition and a shorter clinical course [21]. Thus, in heterozygous cases of sCJD where both PrP^Sc^ with a methionine (PrP^Sc^-M129) and PrP^Sc^ with a valine (PrP^Sc^-V129) may be present, the relative amounts of each allotype (i.e. the PrP^Sc^ allotype ratio), might influence disease pathogenesis. Indeed, there is considerable phenotypic variation within heterozygous cases of MV2 sCJD [3, 23] which might be explained if the PrP^Sc^ allotype ratio influenced disease parameters such as disease incubation time and neuropathology.

Transmission of MV heterozygous cases of CJD into transgenic mice expressing human PrP^C^ would help to further elucidate the role of PrP^Sc^ allotype on disease phenotype. Unfortunately, only a few such studies have been published [1, 2, 5, 20, 23, 25, 44] and most involved the inoculation of only one or two cases of heterozygous sCJD [1, 2, 5, 20, 23]. In one study, inoculation of MV CJD into transgenic mice expressing human PrP^C^-M129 resulted in either short or long incubation times, leading the authors to hypothesize that incubation time might reflect differences in the propagation of PrP^Sc^-M129 versus PrP^Sc^-V129 [2]. However, since it is only recently that mass spectrometry has been used to determine PrP^Sc^ allotype in MV heterozygous cases of CJD [28], the PrP^Sc^ allotype ratio of the MV heterozygous CJD cases transmitted into transgenic mice to date remains unknown. Thus, there is no direct evidence for what effect PrP^Sc^ allotype may have on prion disease incubation time and phenotype.

We have recently shown that the PrP^Sc^ allotype ratio is variable in MV1 and MV2 cases of sCJD but somewhat less variable in cases of MV2K iCJD in the United Kingdom (UK) [28] that were the result of exposure to prion contaminated human growth hormone derived from cadavers. In particular, our analysis of four MV2 sCJD cases showed that statistically significant differences in the amount of PrP^Sc^-M129 isolated from different brain regions of a single patient were associated with brain region-specific differences in PrP^Sc^ deposition [28]. In order to understand the potential contribution of the PrP^Sc^ allotype to disease transmission and phenotype in heterozygous CJD subtypes, we inoculated 10 cases of MV heterozygous sCJD or iCJD into transgenic mice overexpressing human PrP^C^-M129. In one case, brain-region specific differences in PrP^Sc^ allotype appeared to correlate with differences in prion disease transmission and phenotype. Differences in PrP^Sc^ allotype did not consistently correlate with disease incubation times in the other 9 cases but the presence of PrP^Sc^-V129 was associated with plaque deposition. Thus, while our data are consistent with differences in the PrP^Sc^ allotype contributing to variable phenotypes in MV heterozygous cases of CJD, it is not the primary determinant of the final disease phenotype.

## Materials and methods

### Ethics statement

Human brain samples were obtained either from the National CJD Research & Surveillance Unit Brain and Tissue Bank, which is part of the MRC Edinburgh Brain & Tissue Bank (Edinburgh Brain Bank 16-ES-0084), UK, or from the University of Verona, Italy. These latter tissues were obtained at autopsy and sent to the Neuropathology Unit at the University of Verona for statutory definite diagnosis of CJD. Ethical approval for the acquisition and use of post-mortem human CJD brain samples was obtained from the National Institutes of Health (NIH) Office of Human Subject Research (Exempt #11763 and #12725) and no patient identifiable data was transferred to the NIH. The neuropathological and biochemical characteristics of all samples inoculated have been previously described [28].

### Inoculation of tg66 and tgRM mice

The animal experimental protocol was reviewed and approved by the Rocky Mountain Laboratories Animal Care and Use Committee. This study was carried out in strict accordance with the recommendations in the *Guide for the Care and Use of Laboratory Animals* of the National Institutes of Health.

Tg66 and tgRM mice over-expressing human PrP^C^-M129 [37] were infected by injecting 50 μl of brain homogenate from confirmed CJD cases via the intracerebral route using a 27-gauge ½ inch needle. All brain homogenates were prepared using the following procedure. A 10% weight per volume (w/v) solution was prepared from frozen brain tissue in sterile phosphate buffered saline by homogenizing with 2 × 30 s bursts using a Minibeadbeater (Biospec Products). Each sample was sonicated for 2 mins, then further diluted to 1% w/v in phosphate buffered balanced salts solution (PBBS; 1.5 mM potassium phosphate monobasic, 6.6 mM sodium phosphate dibasic anhydrous, 1.3 mM calcium chloride anhydrous, 4.3 mM potassium chloride, 123.2 mM sodium chloride, 1 μM magnesium chloride 6-hydrate, 0.8 μM magnesium sulfate 7- hydrate, 1% phenol red, and 5.6 mM glucose) plus 2% fetal bovine serum. Samples were vortexed and then spun for 1 min at 3000 rpm immediately prior to injection. The titer of the CJD MM1 control was 2 × 10^5.4^ ID_50_/g brain tissue.

Mice were monitored twice weekly for signs of disease. Once early clinical signs appeared, such as aberrant nesting and ruffled fur, mice were checked daily. Late neurological clinical signs included kyphosis, somnolence, reluctance or inability to move normally, ataxia, and visible weight loss. When mice displayed these late clinical signs, they were euthanized using isoflurane in a jar in a scavenger hood. Brains were removed, cut in half along the sagittal midline, and one half was frozen in liquid nitrogen for use in western blot while the other half was placed into 10% normal buffered formalin (NBF) for fixation prior to immunohistochemical analysis.

### Real time quaking induced conversion (RT-QuIC) endpoint titration assay

Brain homogenates were serially diluted (10-fold dilution series from 10^− 3^ to 10^− 10^) in RT-QuIC reaction solution (0.1% sodium dodecyl sulfate (SDS), 1x N-2 supplement (ThermoFisher) and 1x phosphate buffered saline PBS; 130 mM NaCl, 10 mM PO4, pH 7.4). Prion seeding activity for each dilution was measured in a 96-well plate by adding 2 μL diluted brain homogenate to 98 μL RT-QuIC reaction solution to give final concentrations of 0.1 mg/ml bacterially derived and purified recombinant hamster PrP^C^ (amino acid residues 90–231) [29], 10 mM phosphate buffer pH 7.4, 10 μM thioflavin T (ThT), 300 mM NaCl, 1 mM ethylenediaminetetraacetic acid (EDTA) and 0.002% SDS. Four technical replicate reaction wells were prepared for each dilution. The plates were then shaken in a temperature-controlled fluorescence plate reader (BMG FLUOstar) at 50 °C with cycles of 1 min double orbital shaking at 700 rpm and 1 min of rest. ThT fluorescence was measured at 45-min intervals. For each sample, the assay was repeated three times for a total of 12 replicate wells per dilution. The prion seeding dose (SD), defined as the dilution in an endpoint RT-QuIC dilution assay at which 50% of the wells are ThT positive [45], was calculated using the Spearman-Kärber method [17] and reported as SD_50_/g of brain homogenate.

### Immunohistochemistry

Formalin fixed tissue was processed and embedded in paraffin. Sections (5 μm) were cut using a standard Leica microtome, placed on positively charged glass slides, and air-dried overnight at room temperature. Prior to deparaffinization slides were heated in an oven at 60 °C for 20 min. The brain tissue was deparaffinized in Pro-Par clearant (Anatech Innovator), followed by rehydration in decreasing concentrations of ethanol with a final rinse in distilled water. Slides were immersed in 10 mM citrate buffer (pH 6.0), placed in a Biocare decloaking chamber, and treated at 120 °C at 20 lb./in^2^ pressure for 20 min. After cooling, the slides were stained using DABMap kit and hematoxylin counterstain on an automated Discovery XT staining system (Ventana Medical Systems). The primary antibody was the anti-PrP mouse monoclonal antibody 3F4 conjugated to biotin (Covance) diluted 1:50 in Ventana antibody dilution buffer (Ventana Medical Systems) with an incubation time of 60 min.

### Other stains

The presence of amyloid plaques was confirmed by staining deparaffinized sections with the amyloid dye Thioflavin S (ThioS) as previously described [35]. Hamotoxylin and eosin (H&E) staining was performed by hand on deparaffinized slides using Shandon Instant Hemotoxylin and Eosin (Thermo Scientific) according to the manufacturer’s instructions.

### SDS-PAGE and Western blotting

Brain halves from tg66 and tgRM mice were homogenized in PBS to 20% (w/v) with 2 × 30 s bursts using a Minibeadbeater. Tris-HCL (pH 8.5), sodium deoxycholate , and Triton X-100 were added to the homogenate to give final concentrations of 0.1 M, 1%, and 1%, respectively. Samples were protease treated by adding proteinase K (PK) to a final concentration of 63.3 μg/ml followed by incubation for 30 min at 37 °C in a circulating water bath. The PK was then neutralized by adding 0.1 M phenylmethylsulfonyl fluoride (PMSF) to a final concentration of 0.01 M. For each sample, 17.2 μl of Novex NuPage lithium dodecyl sulfate (LDS) 4X sample loading buffer (Life Technologies) was pre-diluted to 2X, 17.2 μl of sample was added, and the sample boiled for 3 min. Samples were loaded onto a 15 well, 1.5 mm NuPage 4–20% Bis-Tris gradient gel (Invitrogen) and run for 75 min at constant voltage (120 V).

After electrophoresis, samples were transferred to polyvinylidene difluoride (PVDF) membrane (Millipore) at 37 V overnight at 4^o^ C in Towbin’s buffer (0.25 M Tris, 1.92 M Glycine, 0.01% SDS, 20% methanol). Membranes were blocked with Blotto (5% milk in Tris buffered saline plus Tween (TBST): 137 mM NaCl, 2.7 mM KCl, 19 mM Tris base, 0.1% Tween 20) for 1.5 h. The membrane was developed using the anti-PrP mouse monoclonal antibody 3F4 conjugated to biotin (Covance) at a 1:10,000 dilution for 1.5 h at room temperature on a shaking platform. Following a 30 min period of 3 to 4 washes in TBST, the membrane was incubated for 1 h at room temperature in a 1:250,000 dilution in TBST of streptavidin-horseradish peroxidase (SA-HRP, Cell Signaling Technology). The wash step above was repeated and the membranes rinsed in distilled water before being developed with SuperSignal West Femto Maximum Sensitivity Substrate (Thermo Scientific) for detection on X-ray film.

### Statistics

The unpaired student’s t-text, Tukey’s multiple comparisons test, linear regression, and sample mean and standard deviation were all calculated using the GraphPad Prism software package (version 8.2).

## Results

### Transmission of MV heterozygous cases of sCJD and iCJD into transgenic mice overexpressing human PrP^C^

We have previously shown that variability in the PrP^Sc^ allotype ratio can be associated with different types of PrP^Sc^ deposition within the brain [28]. In order to determine whether or not the PrP^Sc^ allotype ratio influenced the transmission properties of MV heterozygous cases of CJD, brain samples from 10 cases of codon 129 heterozygous sCJD (*n* = 7) or iCJD (*n* = 3) whose prion disease phenotype had been previously characterized both neuropathologically and biochemically [28], were tested for their ability to transmit disease to transgenic mice overexpressing human PrP^C^-M129. The cases were selected based upon the amount of PrP^Sc^-M129 present in the sample, which ranged from 100 to 29% of the total PrP^Sc^ and represented 4 different neuropathological subtypes of CJD (Tables 1 and 2). All cases had detectable, but variable, levels of PrP^Sc^ as determined by mass spectroscopy (Table 1 and [28]) and western blot [28]. Using the RT-QuIC endpoint titration assay, most of the cases had similar prion seeding activities (Table 1) suggesting that they had similar levels of prion infectivity [45].

**Table 1:** PrP^Sc^ abundance and prion seeding activity in CJD brain homogenate samples inoculated into transgenic mice

Case #^a^	Subtype^a^	PrP^Sc^ (SpC)^b^	SD_50_/g^c^
Control	MM1	ND	> 5 × 10^9^
3	MV1 + 2C	280 ± 103	5 × 10^9^
4	MV1 + 2C	48 ± 9	9 × 10^7^
9 CC	MV2K + 2C	56 ± 12	8.9 × 10^10^
9 CbC	MV2K + 2C	126 ± 15	2.8 × 10^10^
12	MV2K + 2C	90 ± 4	1.6 × 10^9^
19 (iCJD)	MV2K	133 ± 19	3.5 × 10^11^
10	MV2K	175 ± 19	1.2 × 10^11^
20 (iCJD)	MV2K	171 + 44	1.6 × 10^11^
7	MV2K	581 ± 89	2.8 × 10^11^
18 (iCJD)	MV2K	65 ± 7	2.8 × 10^11^
11	MV2C	290 ± 9	3.4 × 10^10^

^a^Neuropathological subtype of cases originally described in [28]. All cases are sCJD except where noted as iCJD. *CC* cerebral cortex, *CbC* cerebellar cortex

^b^*SpC* spectral counts. Total number of PrP^Sc^ peptides as determined by mass spectrometry of phosphotungstic acid precipitated CJD brain material For mean + standard deviation in previous sentence, please underline the + sign. are adapted from [28]

^c^SD_50_/g = prion seeding doses (SD) yielding 50% positive wells in replicate RT-QuIC reactions per 1 g of MV brain homogenate [45]. Values were calculated from 3 independent end-point titration experiments

**Table 2:** Transmission of sCJD and iCJD with variable PrP^Sc^ allotypes into tg66 and tgRM mice overexpressing human PrP^C^-M129

			tg66		tgRM	
Case #^**a**^	Subtype^**b**^	PrP^**Sc**^-M129^**c**^	DPI^**d**^	Clin/total^**e**^	DPI^**d**^	Clin/total^**e**^
Control	MM1	100	187 ± 13	9/9	211 ± 11	8/8
3	MV1 + 2C	100 ± 0****	196 ± 19	8/8	218 ± 13	7/8
4	MV1 + 2C	34 ± 5	> 611	0/7	> 651	0/6
9 (CC)	MV2K + 2C	82 ± 8**	> 502	0/5	> 662	0/7
9 (CbC)	MV2K + 2C	33 ± 8	488 ± 17	5/5	519 ± 26	2/4
12	MV2K + 2C	29 ± 20	497 ± 132	2/5	471	1/7
19 (iCJD)	MV2K	66 ± 4***	184 ± 2	3/3	214 ± 9	7/7
10	MV2K	43 ± 2	407 ± 36	7/7	503 ± 54	5/6
20 (iCJD)	MV2K	39 ± 6	482 ± 34	2/7	373	1/5
7	MV2K	38 ± 18	398 ± 13	6/6	448 ± 18	4/5
18 (iCJD)	MV2K	36 ± 5	238 ± 17	5/8	296 ± 46	4/7
11	MV2C	85 ± 1	401 ± 21	2/8	> 790	0/8

^a^Neuropathological subtype of cases originally described in [28]. All cases are sCJD except where noted as iCJD. *CB* cerebellar cortex, *CC* cerebral cortex

^b^Cases originally described in [28]. MV2K = CJD subtype MV2 with kuru (K) plaques; 2C = Type 2 PrP^Sc^ in the cortex

^c^Percentage of PrP^Sc^ in the inoculum which is PrP^Sc^-M129 shown as mean + standard deviation. Data adapted from [28]. **** *p* = 0.0001 versus case 4 by unpaired student’s t-test; ***p = 0.02 versus case 10, 0.007 versus case 20, 0.009 versus case 7, and 0.003 versus case 18 using Tukey’s multiple comparisons test; ** *p* = 0.01 versus case 9 (CbC) and 12 using Tukey’s multiple comparisons test

^d^DPI = mean days post-infection + standard deviation

^e^Number of mice with clinical disease over total number of mice inoculated. Intercurrent deaths are not included

**Fig. 1:**
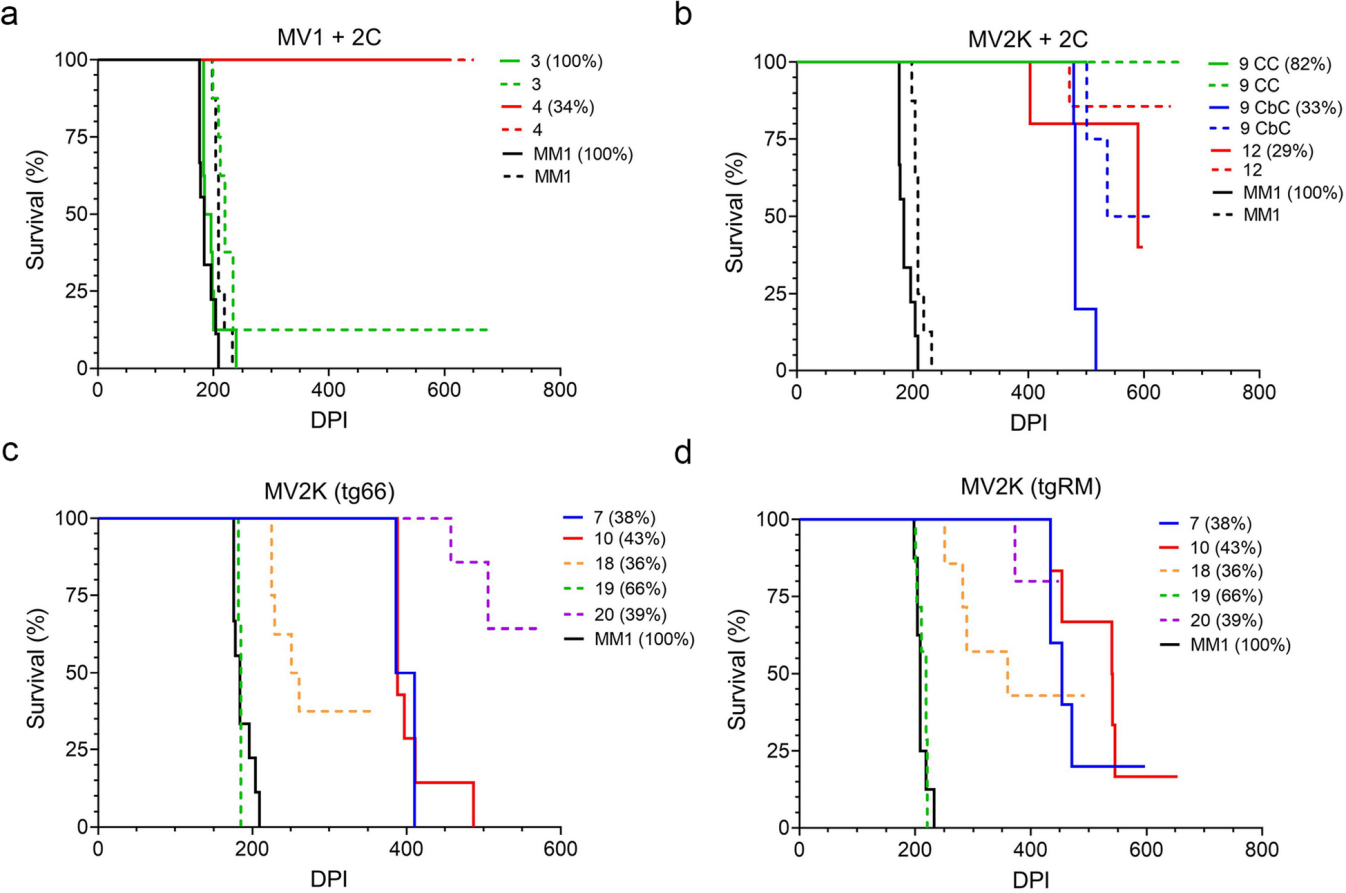
Survival curves for tg66 and tgRM mice inoculated with MV heterozygous CJD brain homogenate containing variable levels of PrP^Sc^-M129. Survival curves for mice inoculated with (**a)** MV1 + 2C or (**b**) MV2K + 2C sCJD. The percentage of PrP^Sc^-M129 for each case inoculated is shown in parentheses to the right of the case number. Solid lines = tg66 mice; dashed lines = tgRM mice. Survival curves for (**c**) tg66 or (**d**) tgRM mice inoculated with MV2K CJD. The percentage of PrP^Sc^-M129 for each case inoculated is shown in parentheses to the right of the case number. Solid lines = sCJD cases; dashed lines = iCJD cases

Brain homogenate samples from the cerebral cortex (CC) or the cerebellar cortex (CbC) were inoculated intracranially into two different strains of transgenic mice over-expressing human PrP^C^-M129 [37], tgRM and tg66. Since both mouse strains are homozygous for PrP^C^-M129, we hypothesized that they would be more susceptible to heterozygous CJD isolates containing higher levels of PrP^Sc^-M129. Transmission results are summarized in Table 2 and survival curves are shown in Fig. 1. For both mouse strains, the majority of cases inoculated caused clinical disease in at least some of the mice. As expected, disease incubation times were generally shorter in the tg66 mice which express higher levels of human PrP^C^-M129 when compared to the tgRM mice [38]. Only two samples, case 4 and the CC sample from case 9, failed to cause any clinical disease within the lifetime of the host, although neuropathology and PrP^Sc^ deposition were observed in a few mice suggesting that clinical disease may have eventually developed in those animals.

### The amount of PrP^Sc^-M129 does not correlate with disease incubation time in tg66 or tgRM mice

**Fig. 2:**
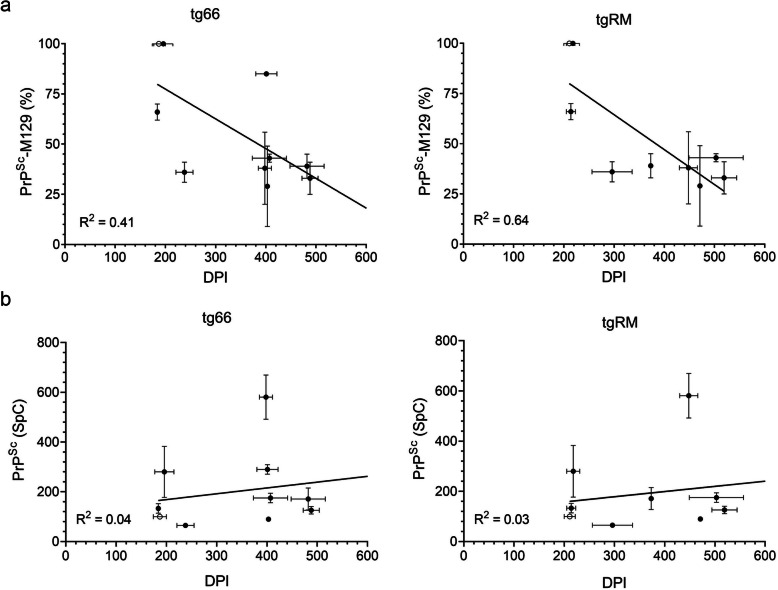
Lack of correlation between PrP^Sc^-M129 or total PrP^Sc^ abundance versus disease incubation time in transgenic mice inoculated with MV heterozygous CJD brain homogenate. **a** The y-axis shows the percentage of total PrP^Sc^ which is PrP^Sc^-M129 versus disease incubation time in days post-infection (DPI) for tg66 (left panel) and tgRM (right panel) mice. Mean + SD is shown for each CJD sample (closed circles) and the MM1 sCJD control (open circle). The results of the linear regression analysis are represented by the line and the R^2^ value is given in the lower left corner of each graph. **b** The y-axis shows the spectral count (SpC) of the total PrP^Sc^ in the sample versus disease incubation times in DPI for tg66 (left panel) and tgRM (right panel) mice. Figure legend and linear regression analysis are the same as in panel **a**

Consistent with previous transmission studies of heterozygous CJD cases [1, 2, 5, 23, 25], extended disease incubation times of more than 400 days were observed for most of the samples tested, although in many instances clinical signs of disease were observed in only a percentage of the total mice inoculated (Fig. 1 and Table 2). The exceptions were cases 3, 18, and 19 which caused clinical disease with incubation times of less than 400 days. In cases 3 and 19, PrP^Sc^-M129 comprised 100 and 66%, respectively, of the total PrP^Sc^ while in case 18 PrP^Sc^-M129 comprised only 36% of the total PrP^Sc^. Thus, our transmission results would appear to be inconsistent with higher levels of PrP^Sc^-M129 generally being predictive of faster disease incubation times. Indeed, two other MV heterozygous CJD samples with greater than 80% PrP^Sc^-M129 either did not cause disease (case 9, CC sample) or caused disease in only a minority of tg66 mice inoculated (case 11). A linear regression analysis comparing clinical disease incubation times to PrP^Sc^-M129 levels confirmed that, for both mouse strains, there was a poor correlation between the amount of PrP^Sc^-M129 in the heterozygous CJD sample and clinical disease incubation time (Fig. 2a). In addition, we found no linear correlation of total PrP^Sc^ levels in the brain with clinical disease incubation time in either tg66 or tgRM mice (Fig. 2b). Thus, our data suggest that the observed differences in disease incubation time were unrelated to either PrP^Sc^-M129 or total PrP^Sc^ levels.

### CJD PrP^Sc^ type is not maintained following transmission into tg66 and tgRM mice

**Fig. 3:**
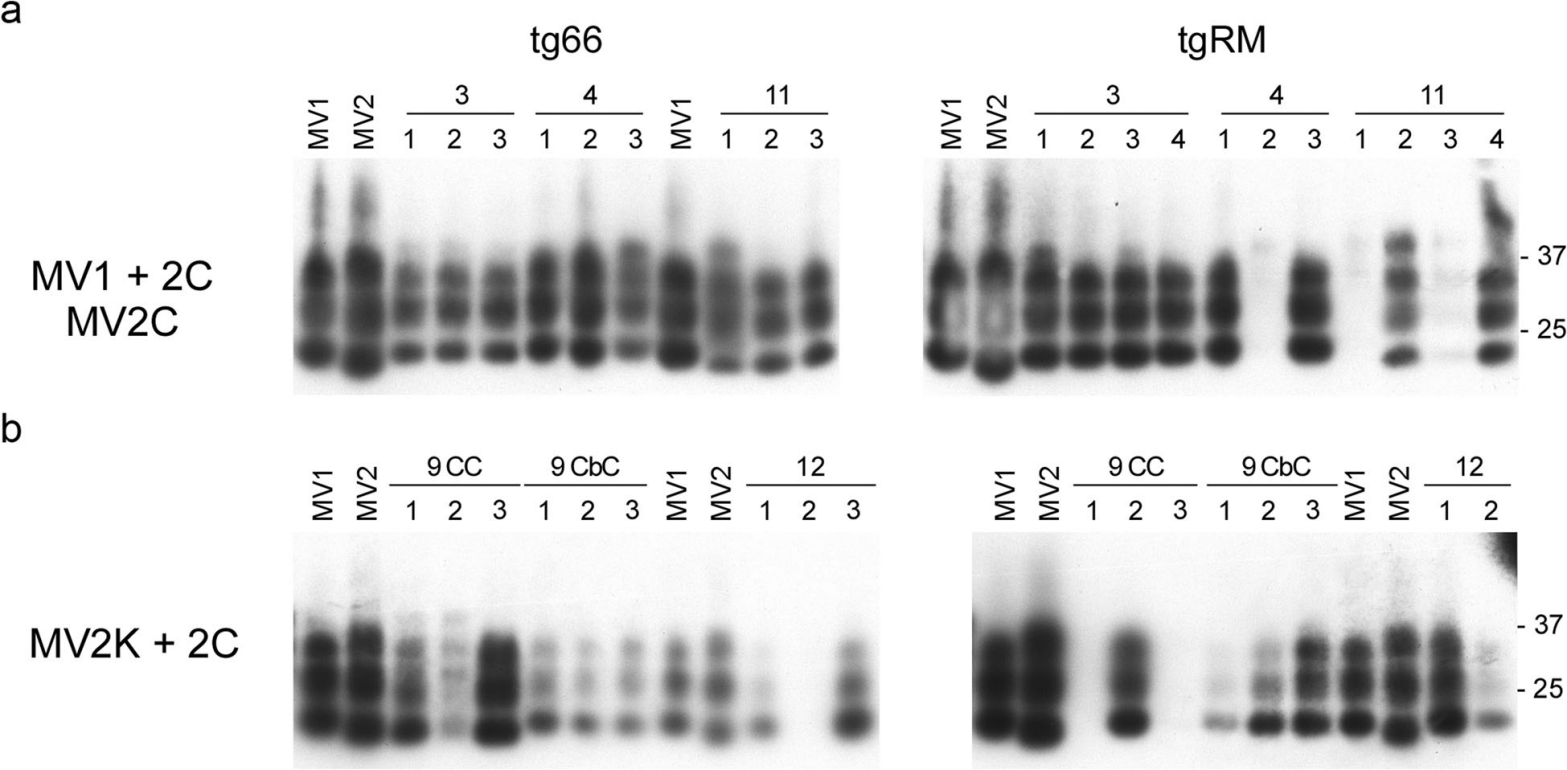
Type 1 PrP^Sc^ accumulates in the brains of most tg66 and tgRM mice inoculated with 3 different subtypes of MV heterozygous sCJD. **a** PrP^Sc^ immunoblot of tg66 mice (left panel) and tgRM mice (right panel) inoculated with either MV1 + 2C (cases 3 and 4) or MV2C (case 11) sCJD. Type 1 PrP^Sc^ accumulates in all of the recipient mice except for those inoculated with MV2C case 11 which accumulate Type 2 PrP^Sc^. The numbers above the line indicate the case number while the numbers over the individual lanes represent the 3–4 brain samples that were analyzed for each CJD subtype inoculated. **b** PrP^Sc^ immunoblot of tg66 mice (left panel) and tgRM mice (right panel) inoculated with MV2K + 2C sCJD. Type 1 PrP^Sc^ accumulates in all of the recipient mice except for tg66 mice inoculated with case 9 (CC) which accumulate Type 2 PrP^Sc^. The numbers above the line indicate the case number while the numbers over the individual lanes represent the 2–4 brain samples that were analyzed for each CJD subtype inoculated. Blots were developed using the mouse monoclonal anti-PrP antibody 3F4 conjugated to biotin. Lanes marked MV1 and MV2 are from human cases of sCJD and represent the controls for Type 1 and Type 2 PrP^Sc^, respectively. Molecular mass markers are indicated on the right side of the figure

**Fig. 4:**
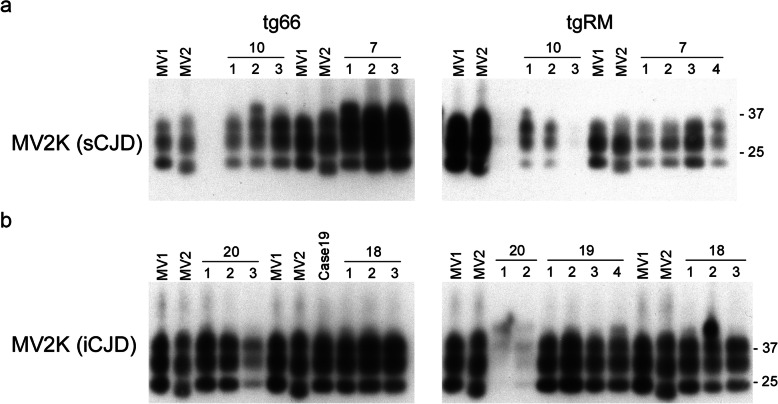
Type 1 PrP^Sc^ accumulates in the brains of tg66 and tgRM mice inoculated with heterozygous cases of MV2K sporadic and iatrogenic CJD. **a** PrP^Sc^ immunoblot of tg66 mice (left panel) and tgRM mice (right panel) inoculated with MV2K cases of sCJD. Type 1 PrP^Sc^ accumulates in all of the recipient mice. The numbers above the line indicate the case number while the numbers over the individual lanes represent the 3–4 brain samples that were analyzed for each CJD subtype inoculated. Lanes with no label above them are blank. **b** PrP^Sc^ immunoblot of tg66 mice (left panel) and tgRM mice (right panel) inoculated with MV2K cases of iCJD. Type 1 PrP^Sc^ accumulates in all of the recipient mice. The numbers above the line indicate the case number while the numbers over the individual lanes represent the 3–4 brain samples that were analyzed for each CJD subtype inoculated except for case 19 where only one sample was available for the tg66 mice. Blots were developed using the mouse monoclonal anti-PrP antibody 3F4 conjugated to biotin. Lanes marked MV1 and MV2 are from human cases of sCJD and represent the controls for Type 1 and Type 2 PrP^Sc^, respectively. Molecular mass markers are indicated on the right side of the figure

We next looked at the size of PrP^Sc^ deposited in the brain. Previous studies have shown that inoculation of MM1, MV1, and MV2 cases of CJD into mice homozygous for PrP^C^-M129 always resulted in the deposition of Type 1 PrP^Sc^ in the brains of recipient mice [5, 23, 25]. We also found that Type 1 PrP^Sc^ accumulated in tg66 and tgRM mice inoculated with both the MV1 (Fig. 3) and MV2K (Fig. 4) CJD subtypes. However, for two samples that did not transmit well to mice, cases 11 and 9 CC, Type 2 PrP^Sc^ accumulated in at least one of the two recipient mouse lines inoculated (Fig. 3). While these data are consistent with Type 2 PrP^Sc^ inhibiting disease, it is important to note that accumulation of Type 1 PrP^Sc^ was also observed in cases that transmitted poorly (Fig. 3a and Fig. 4, see cases 4 and 20). Thus, our results suggest that the type of PrP^Sc^ deposited in the brain does not appear to correlate well with prolonged disease incubation times and/or lack of clinical disease. Moreover, they support a previous study [5] which suggested that the final PrP^Sc^ type in recipient mice is not determined solely by the PrP^Sc^ type in the inoculum but rather by interactions between the prion strain inoculated and the host.

### PrP^Sc^ allotype and disease phenotype in MV1 + 2C sCJD

Previous studies have shown that efficient transmission of CJD into transgenic mice is influenced by the neuropathological subtype of CJD as well as by whether or not the inoculated mice express PrP^C^-M129 and/or PrP^C^-V129 [1, 2, 5, 9, 13, 23, 44]. Four different CJD subtypes were represented in the samples tested. We therefore analyzed whether, within a given CJD subtype, PrP^Sc^-M129 was potentially influencing transmission into tg66 and tgRM mice. Since only a single case of MV2C was available (Table 2), this subtype was not included in the analysis.

Sporadic CJD subtype MV1 + 2C, which is neuropathologically similar to MM1 sCJD [33], was represented by two cases that differed significantly in the amount of PrP^Sc^-M129 present (Table 2). Case 3, where PrP^Sc^-M129 represented 100% of the total PrP^Sc^, caused clinical disease when inoculated into both tg66 and tgRM mice with incubation times indistinguishable from that of MM1 sCJD (Table 2 and Fig. 1a). Case 4, where PrP^Sc^-M129 represented only 34% of the total PrP^Sc^, did not cause clinical disease in either mouse strain (Table 2 and Fig. 1a). It was possible that the slightly higher seeding activity of case 3 (Table 1) accounted for its shorter incubation time and higher disease transmissibility. However, since cases 3 and 4 were classified as the same histopathological subtype [28], the starting seeding activity or titer should not influence the final neuropathological profile and pattern of PrP^Sc^ deposition. We therefore analyzed the neuropathological features of both cases following transmission into transgenic mice. In general, the neuropathological features were similar in both tg66 and tgRM mice (Supplementary Table 1), and only results from tg66 mice are shown for most of the CJD subtypes inoculated.

**Fig. 5:**
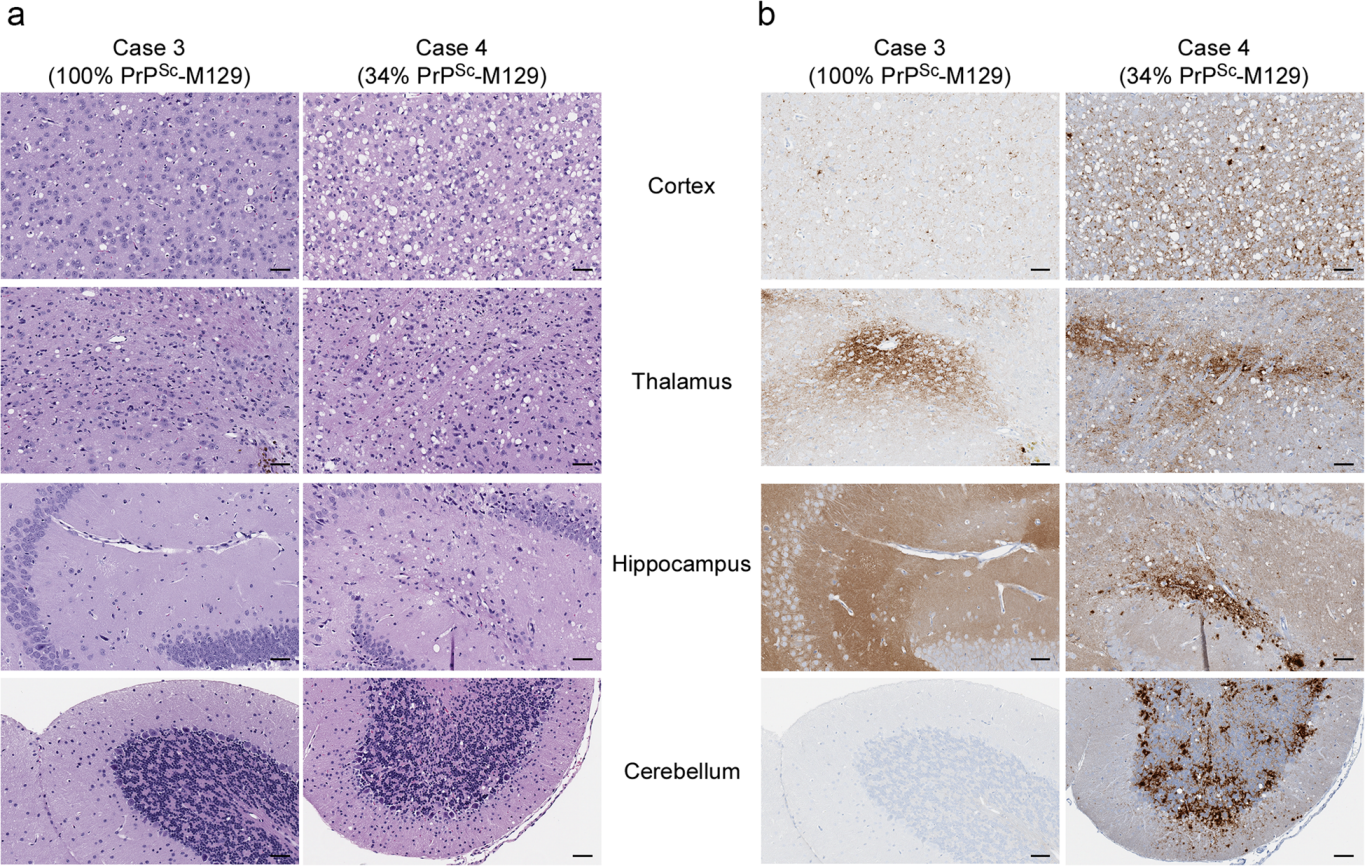
Spongiform change and PrP^Sc^ deposition differ in the brains of tg66 mice inoculated with cases of MV1 + 2C with different PrP^Sc^ allotype ratios. **a** H&E staining of tg66 mice inoculated with MV1 + 2C cases 3 (183 dpi) and 4 (479 dpi). The mean percentage of PrP^Sc^-M129 in each sample is given under the case number. **b** PrP^Sc^ staining using the mouse monoclonal anti-PrP antibody 3F4 conjugated to biotin. The fields are matched to those shown in panel **a**. The brain regions shown are indicated in the middle of the figure. For all panels, scale bar = 50 μm

Spongiform change was present in the hippocampus and granular layer of the cerebellum and was more severe in the cortex in mice inoculated with case 4 when compared to case 3, which had less extensive vacuolation in the cortex and no spongiform change apparent in either the cerebellum or hippocampus (Fig. 5a). In terms of PrP^Sc^ deposition, diffuse, punctate/synaptic PrP^Sc^ was deposited in mice inoculated with both cases (Fig. 5b), but only case 4 had occasional plaque-like deposits as well as dense PrP^Sc^ deposits in the hippocampus and granular layer of the cerebellum (Fig. 5b). In addition, both cases were clearly neuropathologically distinct from MM1 sCJD which was characterized by mild to moderate spongiform change and diffuse, synaptic/punctate deposition of PrP^Sc^ in both mouse lines. (Supplementary Table 1, Supplementary Fig. 1 and Fig. 2). Thus, the data suggest that the prions in cases 3 and 4 have distinct properties that differ not only from each other but also from the MM1 subtype of CJD prions to which they are neuropathologically similar [33]. Our data show a lack of consistency in transmission properties of cases of MV1 + 2C sCJD where the abundance of PrP^Sc^-M129 is significantly different and suggest that there is greater phenotypic variability within this CJD subtype than has been previously recognized.

### PrP^Sc^ allotype and disease in MV2K sporadic and iatrogenic CJD

**Fig. 6:**
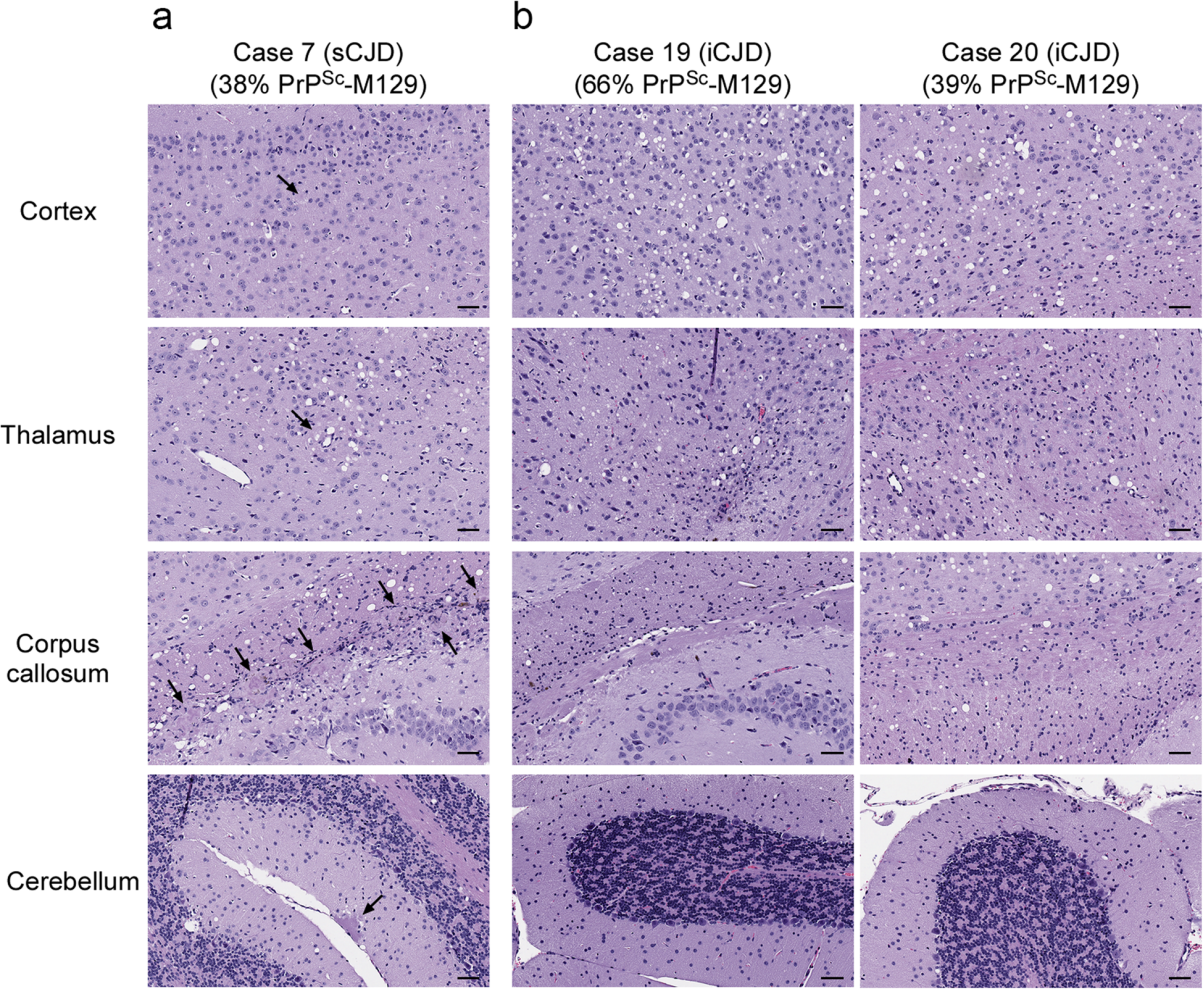
Variable spongiform change and amyloid plaque deposition in the brains of tg66 mice inoculated with sporadic or iatrogenic cases of MV2K CJD. **a** H&E staining of a tg66 mouse inoculated with MV2K sCJD case 7 (386 dpi). The black arrows indicate eosinophilic amyloid plaques. The neuropathological features of case 10 were identical to those of case 7. **b** H&E staining of tg66 mice inoculated with MV2K iCJD cases 19 (185 dpi, left column) and 20 (458 dpi, right column). No eosinophilic amyloid plaques are detectable in any of the brain regions shown. The neuropathological features of case 18 were the same as those of case 19. The mean percentage of PrP^Sc^-M129 in each sample is given under the case number. The brain regions shown are indicated on the left. For all panels, scale bar = 50 μm

**Fig. 7:**
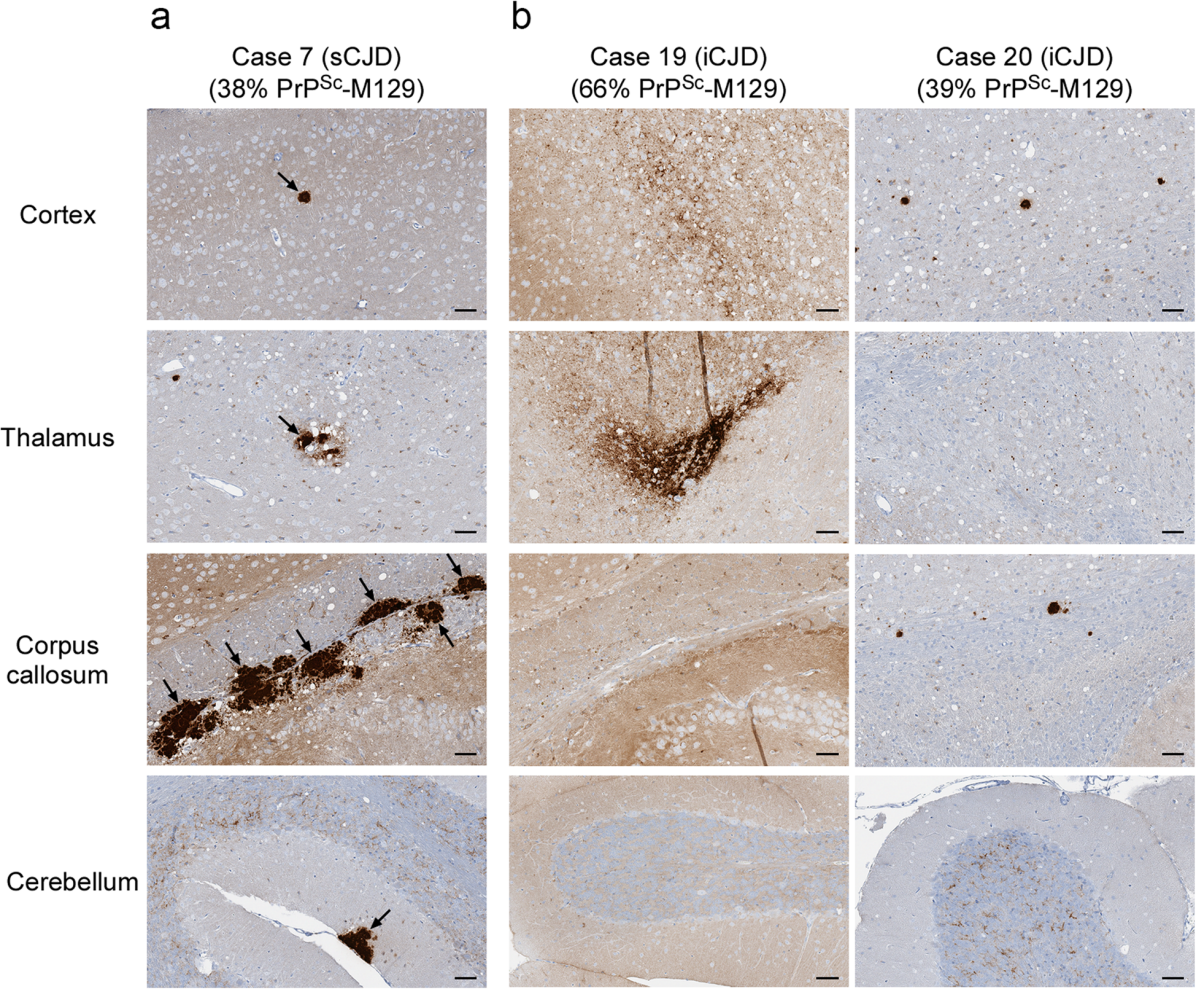
Different patterns of PrP^Sc^ deposition in the brains of tg66 mice inoculated with sporadic or iatrogenic cases of MV2K CJD. **a** PrP^Sc^ deposition in a tg66 mouse inoculated with MV2K sCJD case 7 (386 dpi). The black arrows indicate PrP^Sc^-positive amyloid plaques. The fields are matched to those shown in Fig. 6a. PrP^Sc^ deposition in case 10 was identical to that of case 7. **b** PrP^Sc^ deposition in tg66 mice inoculated with MV2K iCJD cases 19 (185 dpi, left column) and 20 (458 dpi, right column). The fields are matched to those shown in Fig. 6b. PrP^Sc^-positive amyloid plaques are not present but occasional plaque-like deposits of PrP^Sc^ were observed. PrP^Sc^ deposition in case 18 was the same as that of case 19. The mean percentage of PrP^Sc^-M129 in each sample is given under the case number. All sections were stained using the mouse monoclonal anti-PrP antibody 3F4 conjugated to biotin. For all panels, scale bar = 50 μm

We next analyzed whether the PrP^Sc^ allotype ratio correlated with the transmission properties of the 5 cases of MV2K inoculated. The two sporadic CJD MV2K cases, which contained similar levels of PrP^Sc^-M129 (Table 2), caused clinical disease with similar incubation times in all of the tg66 mice (Table 2, Fig. 1c) and the majority of tgRM mice inoculated (Table 2, Fig. 1d). Pathologically, the two cases were indistinguishable and were characterized by mild to moderate spongiform change in both mouse strains particularly in the thalamus and corpus callosum (Supplementary Table 1, Fig. 6a). PrP^Sc^ deposition was characterized mainly by a mix of perivacuolar PrP^Sc^ and scattered PrP^Sc^ amyloid plaques in many areas of the brain including very large plaques in the corpus callosum (Fig. 7a).

The other three MV2K samples inoculated were human growth hormone-related cases of iatrogenic CJD from the UK. Unexpectedly, these cases differed in their presentation from the sCJD MV2K samples inoculated. Case 19, which had significantly more PrP^Sc^-M129 than the other 4 MV2K cases (Table 2), was also the only MV2K sample which caused clinical disease in all tgRM and tg66 mice inoculated (Fig. 1c and d) with incubation times indistinguishable from that of MM1 sCJD (Table 2). Despite the different disease incubation times, the neuropathological features for MV2K cases 18 and 19 were similar and distinct from that of the two sCJD MV2K cases. Mild to moderate spongiform change was observed in most regions of the brain (Fig. 6b) and PrP^Sc^ was deposited in a diffuse, punctate/synaptic pattern (Fig. 7b) with amyloid plaques limited to the area around the needle scar (Supplementary Fig. 3). Interestingly, PrP^Sc^ deposition in case 20 differed somewhat in that synaptic PrP^Sc^ deposition was less prominent and plaque-like deposits were observed in multiple brain regions (Fig. 7b). The combined transmission data from the 5 MV2K samples are consistent with the possibility that a greater abundance of PrP^Sc^-M129 correlates with faster disease incubation times in this CJD subtype. The data also show that prions from sporadic versus iatrogenic cases of MV2K have different properties, suggesting that prions from iatrogenic cases of MV2K CJD are either not derived from sporadic MV2K prions or were altered by passage through humans.

### Brain regions from a single patient which differ in PrP^Sc^ allotype have prions with different transmission properties

In our previous study, we found that the PrP^Sc^ allotype ratio could differ significantly between brain regions within the same patient [28] and that these differences were associated with different patterns of PrP^Sc^ deposition within the brain [28]. In order to determine whether or not the transmission properties of the prions in these brain regions also differed, we inoculated two different brain regions from a single MV2K + 2C patient (case 9) into tg66 and tgRM mice. The cerebral cortex sample from case 9 (9 CC) did not cause clinical disease in either tg66 or tgRM mice (Table 2, Fig. 1b) even though it had a statistically higher abundance of PrP^Sc^-M129 when compared to the other MV2K + 2C samples inoculated. By contrast, a cerebellar cortex sample from the same case (9 CbC) caused clinical disease in all tg66 mice (Table 2, Fig. 1b) and in 50% of the tgRM mice inoculated, even though it had a lower abundance of PrP^Sc^-M129 (Table 2). These results are the opposite of those for cases 3 and 19 where significantly higher levels of PrP^Sc^-M129 relative to PrP^Sc^-V129 were associated with faster disease incubation times (Table 2). Thus, our data suggest not only that prions in two different brain regions from the same patient have different transmission properties but also that the abundance of PrP^Sc^-M129 in heterozygous cases of CJD is not a reliable predictor of disease incubation time.

**Fig. 8:**
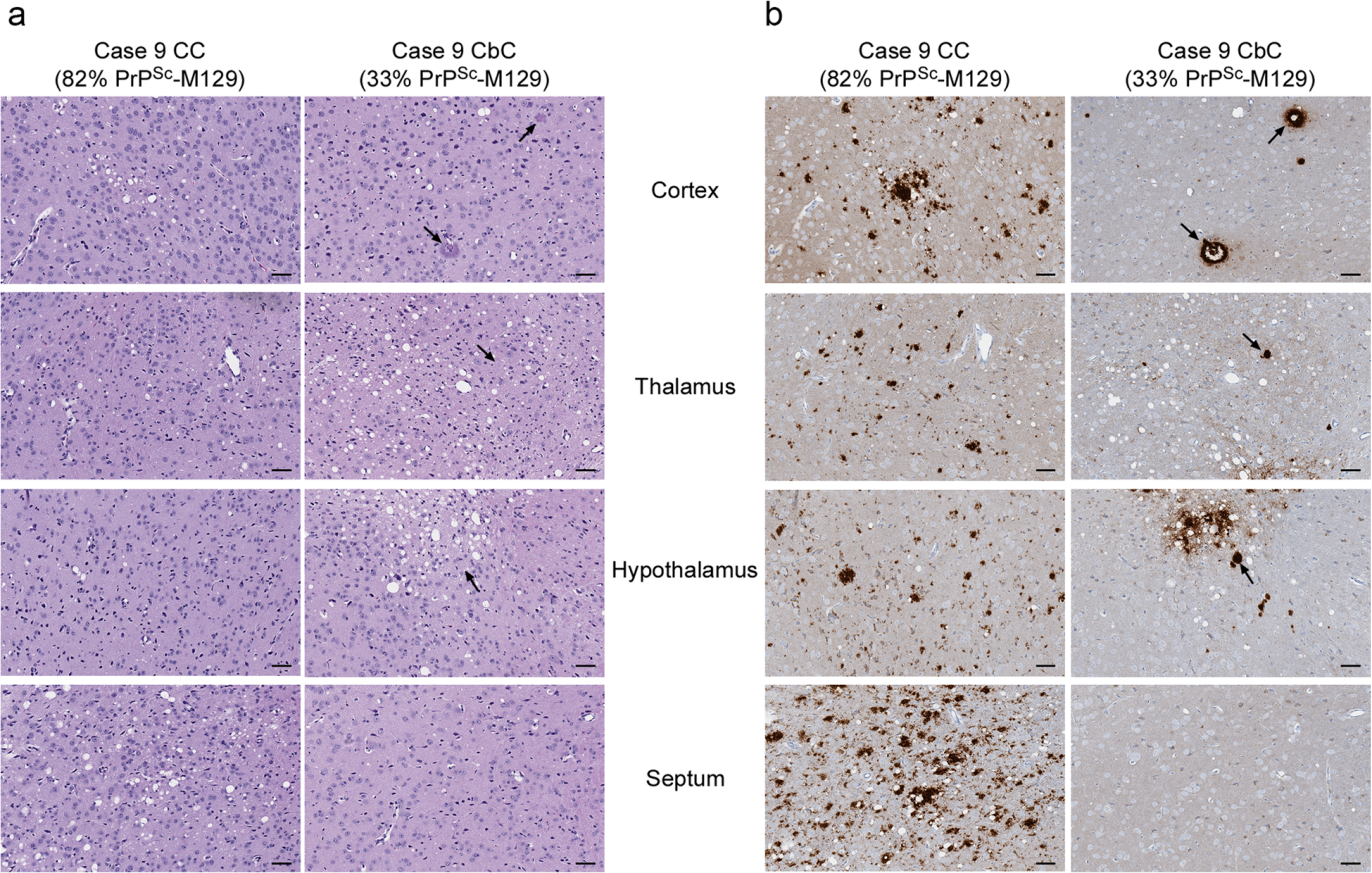
Spongiform change and PrP^Sc^ deposition differ in the brains of tg66 mice inoculated with two different brain regions from a single case of MV2K + 2C sCJD. **a** H&E staining of a tg66 mouse inoculated with MV2K + 2C sCJD case 9 CC (502 dpi, left column) or 9 CbC (481 dpi, right column). The black arrows indicate eosinophilic amyloid plaques. **b** PrP^Sc^ deposition in a tg66 mouse inoculated with MV2K + 2C sCJD case 9 CC (502 dpi, left column) or 9 CbC (481 dpi, right column). The black arrows indicate PrP^Sc^-positive amyloid plaques. The fields are matched to those shown in panel a. Sections were stained using the mouse monoclonal anti-PrP antibody 3F4 conjugated to biotin. The mean percentage of PrP^Sc^-M129 in each sample is given under the case number. Brain regions shown are indicated in the middle of the figure. For all panels, scale bar = 50 μm

Interestingly, samples 9 CC and 9 CbC also differed neuropathologically. Spongiform change tended to be more severe in the mice inoculated with the CbC sample (Supplementary Table 1, Fig. 8a). In addition, PrP^Sc^ deposition and amyloid plaques were more prevalent in the cortex, thalamus, and hypothalamus in the 9 CbC sample when compared to the CC sample where PrP^Sc^ was primarily found in dense, perivacuolar deposits (Supplementary Table 1, Fig. 8b). The prevalence of amyloid plaques in transgenic mice inoculated with the cerebellar sample from case 9 is reflective of what was observed in the original human brain tissue where plaques were prominent in cerebellar tissue but not in cortical tissue [28]. The most striking difference between the two samples was in the septum where spongiform change and dense, perivacuolar deposits of PrP^Sc^ were present in mice inoculated with the CC sample but were completely absent in mice inoculated with the CbC sample (Fig. 8a and b). The transmission and neuropathological data together strongly suggest that, in heterozygous cases of sCJD, prions with different infectious properties can arise in brain regions with different PrP^Sc^ allotypes.

A third MV2K + 2C sample was inoculated that had different transmission properties when compared to the two samples from case 9. Case 12 caused disease in at least some tg66 and tgRM mice inoculated but only after a prolonged incubation time (Table 2, Fig. 1b). Strikingly, the neuropathology and PrP^Sc^ deposition induced by the case 12 sample varied depending upon the mouse strain. In tgRM mice, mild spongiform change was observed (Supplementary Table 1, Supplementary Fig. 4a) while diffuse PrP^Sc^ deposition was observed along with large amyloid plaques (Supplementary Fig. 4b). By contrast, in the single tg66 mouse where pathology was present, spongiform change was more extensive (Supplementary Table 1, Supplementary Fig. 4b) and PrP^Sc^ was deposited in a diffuse, synaptic pattern with no amyloid plaques apparent (Supplementary Fig. 4b). Overall, our data show that there can be considerable variability in the transmission properties of MV2K + 2C prions. Moreover, they suggest prions with different transmission properties exist even within well-defined neuropathological subtypes of CJD and that host factors other than PrP^C^ sequence can significantly influence the final prion disease phenotype.

## Discussion

In transgenic mice expressing only human PrP^C^-M129, a mismatch at amino acid 129 in the inoculum can influence disease incubation time. Thus, the MM1 subtype of sCJD transmits most efficiently into transgenic mice homozygous for human PrP^C^-M129 [2, 5, 25, 43]. However, transmission of MV heterozygous cases of CJD can vary by incubation time and CJD type [5, 20, 23, 25, 44]. Our hypothesis was that the variable transmission efficiency of heterozygous cases of sCJD into transgenic mice expressing human PrP^C^-M129 was determined by the PrP^Sc^ allotype ratio in the sample inoculated. Our expectation was therefore that MV heterozygous CJD samples with statistically higher levels of PrP^Sc^-M129 would transmit disease more efficiently into our mice than samples where PrP^Sc^-V129 predominated. This hypothesis was supported by data from two of the sCJD subtypes we tested, MV1 + 2C and MV2K, but did not hold true for the MV2K + 2C subtype. In the latter subtype, a greater abundance of PrP^Sc^-M129 in the sample relative to PrP^Sc^-V129 led to prolonged incubation times and had a negative effect on disease transmission. Thus, our data suggest that the relative amount of PrP^Sc^-M129 in cases of heterozygous CJD is not predictive of disease incubation time even when the amino acid at codon 129 is the same in both the inoculum and host.

Earlier studies have suggested that a methionine or valine at codon 129 influences how PrP^Sc^ is deposited [14, 16, 21, 27, 42]. A valine at residue 129 is associated with plaque formation and a longer clinical disease course [21] while a methionine at residue 129 is associated with a more synaptic pattern of PrP^Sc^ deposition and shorter clinical course [21]. Our transmission data suggest that PrP^Sc^ allotype ratio may impact the pattern of PrP^Sc^ deposition in a similar way. In mice inoculated with samples consisting entirely of PrP^Sc^-M129, PrP^Sc^ was deposited in a diffuse, punctate/synaptic pattern with no amyloid plaques detected. In the MV heterozygous CJD cases where PrP^Sc^-V129 was present, amyloid plaques were detected but their prevalence and location varied. In samples where PrP^Sc^-V129 comprised the majority of total PrP^Sc^, amyloid plaques and plaque-like deposits were more common and more widely distributed than in samples where PrP^Sc^-V129 represented less than half of the total PrP^Sc^ in the samples. In these latter samples, large plaques were found almost exclusively around the needle scar (Supplementary Fig. 3) suggesting a possible lack of spread from the site of inoculation. Thus, we hypothesize that, in MV heterozygous cases of CJD, plaque phenotype and plaque load may reflect the relative abundance of PrP^Sc^-V129 present.

Support for this hypothesis was provided by the pattern of PrP^Sc^ deposition in mice inoculated with two different brain regions from a case of MV2K + 2C. PrP^Sc^ in the cerebellar cortex sample 9 CbC was composed primarily of PrP^Sc^-V129 and led to PrP^Sc^ amyloid plaques as well as perivacuolar PrP^Sc^ deposition in multiple brain regions of the recipient mice. By contrast, PrP^Sc^ in the cerebral cortex sample 9 CC was composed primarily of PrP^Sc^-M129 and was associated mainly with synaptic and perivacuolar PrP^Sc^ deposition (Fig. 8b) with dense plaques found only around the needle scar (Supplementary Fig. 3). These findings were consistent with the pattern of PrP^Sc^ deposition in the original brain material from case 9 [28]. In that case, PrP^Sc^-V129 was significantly more abundant in the cerebellum where plaques were a primary feature of PrP^Sc^ deposition while PrP^Sc^-M129 was significantly more abundant in the cortex where PrP^Sc^ deposition was primarily synaptic [28].

The CC and CbC samples from case 9 led to patterns of PrP^Sc^ deposition in mice that differed from each other but were similar to that in the brain of the original patient. This strongly suggests that prions with distinctive properties, i.e. prion strains, are being propagated in different regions of the brain. Our data are also consistent with the fact that multiple strains of prions have been isolated from a single brain [4, 15]. However, the long incubation time of sCJD makes it difficult to determine how different brain regions might give rise to different prion strains. One possibility is that, if a single spontaneous misfolding event were responsible for the initiation of sCJD, different selective pressures in the cortex versus the cerebellum could select for different prion strains. An alternative possibility is that more than one spontaneous misfolding event occurred, one in the cortex and one in the cerebellum, each of which gave rise to different prion strains. While admittedly speculative, the latter scenario raises the intriguing possibility that spontaneous formation of PrP^Sc^ may not necessarily be a rare event in some individuals but rather a consequence of some other deficit in the protein folding/unfolding machinery that predisposes them to accumulating misfolded protein.

Previous studies have suggested that MV2K and MV2K + 2C sCJD act similarly when transmitted into transgenic mice expressing human PrP^C^-M129. However, these studies were based on the results from a single case of each type [23]. The current study, where 3 samples of MV2K + 2C and 5 samples of MV2K CJD were tested, suggests a more complex picture. As discussed above, we observed phenotypic variability even within a particular CJD subtype. In the MV2K + 2C subtype, prions from two different brain regions of case 9 differed in their transmission phenotype while the pattern of PrP^Sc^ deposition in case 12 differed between tg66 and tgRM mice (Supplementary Fig. 4). This variation in the transmission properties of different cases of MV2K + 2C makes it difficult to compare in any meaningful way with MV2K cases of CJD. In these latter cases, there was variation in disease phenotype depending upon whether they were from sporadic or iatrogenic CJD. This variability suggests that transmission of different isolates of MV2K and MV2K + 2C into our transgenic mice may have led to the propagation of different prion strains. However, further passage in mice will be needed to determine whether or not the phenotypes we observed are both stable and faithfully propagated, key characteristics of prion strains. Overall, the variability we observed within and between subtypes that have been reported to be very similar again suggests a more complex picture of CJD transmission where CJD subtype, PrP^Sc^ conformation, PrP^Sc^ allotype, and the host likely all contribute to the final disease phenotype.

In the UK, the *PRNP* codon 129 genotype of the cohort of iCJD patients associated with exposure to prion-contaminated human growth hormone extracts is primarily MV heterozygous or VV homozygous, with only a small percentage of the patients being MM homozygous [6, 12, 40]. This *PRNP* genotype distribution is distinct from that of sCJD in the UK [6] as well as cohorts of human growth hormone-related iCJD in France [6] and the US [7]. Based on these genotype differences, it has been suggested that UK human growth hormone-related iCJD was the result of exposure to a different prion strain [6, 7] than that responsible for human growth hormone related iCJD in France and the US.It was further proposed that either MV or VV prions were responsible for the UK cases [39, 40], with VV2 prions considered to be the most likely source [39]. Our results demonstrating that the transmission phenotype of MV2K prions from sCJD and iCJD differ would seem to argue against MV2K prions as being the source of infection for human growth hormone-related iCJD in the UK. Nonetheless, it’s still possible that either VV2 prions were the source of infection or that passage through humans altered the properties of the original infectious prions leading to an iCJD MV2K prion strain or strains that do not recapitulate the sCJD MV2K phenotype when inoculated into transgenic mice over-expressing human PrP^C-^M129.

## Conclusions

PrP^Sc^ allotype is not predictive of disease incubation time and is not the primary determinative driver of phenotype in heterozygous cases of CJD. However, it does appear to correlate with the type of PrP^Sc^ deposited. Thus, when PrP^Sc^-V129 predominates, PrP^Sc^ plaques are common. We further conclude that, within a single patient brain, prions with different infectious properties can arise in brain regions with different PrP^Sc^ allotypes. These data may help to explain why multiple prion strains can be isolated from a single brain. Finally, we conclude that MV2K prions from sCJD differ from iCJD MV2K prions, suggesting that sCJD MV2K may not have been the source of infection in human growth hormone related cases of iCJD in the UK. Overall, our data demonstrate that there is more heterogeneity in the transmission properties of CJD neurological subtypes than has been previously described and suggest a complex picture of CJD transmission where CJD subtype, PrP^Sc^ conformation, PrP^Sc^ allotype, and the host likely all contribute to the final disease phenotype.

## Supplementary information

**Additional file 1.** Histopathological and neuropathological characteristics of tg66 and tgRM mice inoculated with either MM1 sCJD or MV heterozygous cases ofsCJD and iCJD with variable PrPSc allotypes.

## Data Availability

Data sharing not applicable to this article as no datasets were generated or analysed during the current study.

## Abbreviations

PrP^C^: normal prion protein
PrP^Sc^: infectious prion protein
BSE: Bovine Spongiform Encephalopathy
NaCL: sodium chloride
KCL: potassium chloride
PO_4_: phosphate
ThT: Thioflavin T
SD: seeding dose
sCJD: sporadic Creutzfeldt-Jakob Disease
iCJD: iatrogenic Creutzfeldt-Jakob Disease
CC: Cerebral cortex
CbC: Cerebellar cortex
RT-QuIC: Real Time Quaking Induced Conversion
SpC: Spectral counts
DPI: Days post-infection
NBF: Normal buffered formalin
PBBS: Phosphate buffered balanced salts solution
PBS: Phosphate buffered saline
SDS: Sodium dodecyl sulfate
EDTA: Ethylenediaminetetraacetic acid
H&E: Hamotoxylin and eosin
ThioS: Thioflavin S
PK: Proteinase K
PMSF: Phenylmethylsulfonyl fluoride
LDS: Lithium dodecyl sulfate
TBST: Tris buffered saline plus Tween
