# Supplementary material for: Transmission characteristics of heterozygous cases of Creutzfeldt-Jakob disease with variable abnormal prion protein allotypes
Source: Acta Neuropathol Commun. 2020 Jun 9;8:83. doi: 10.1186/s40478-020-00958-x (PMC7285538; doi:10.1186/s40478-020-00958-x)
Supplement: Supplementary file 1 — Additional file 1. Histopathological and neuropathological characteristics of tg66 and tgRM mice inoculated with either MM1 sCJD or MV heterozygous cases ofsCJD and iCJD with variable PrPSc allotypes. [file 40478_2020_958_MOESM1_ESM.pdf]

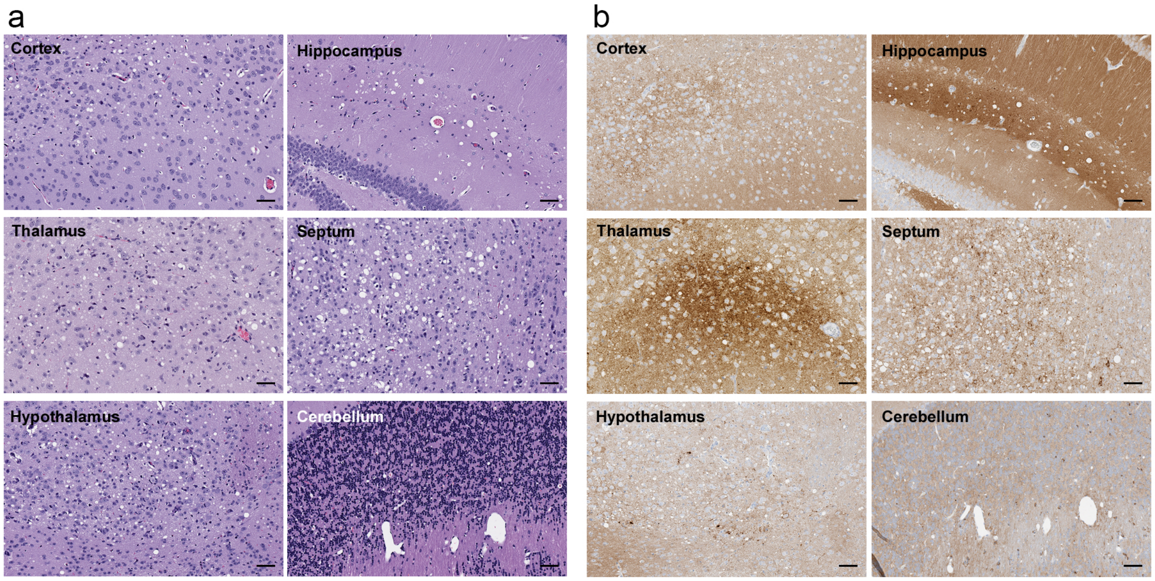

Supplementary Fig. 1 Spongiform change and PrP^{Sc} deposition in tg66 mice inoculated with MM1 sCJD a) H&E staining of tg66 mice inoculated with MM1 sCJD (184 dpi). b) PrP^{Sc} deposition in tg66 mice inoculated with MM1 sCJD. The fields shown are matched to those in panel a. Sections were stained using the mouse monoclonal anti-PrP antibody 3F4 conjugated to biotin. For both panels, the brain region shown is indicated in the upper left-hand corner of each panel. For all panels, scale bar = 50 microns.

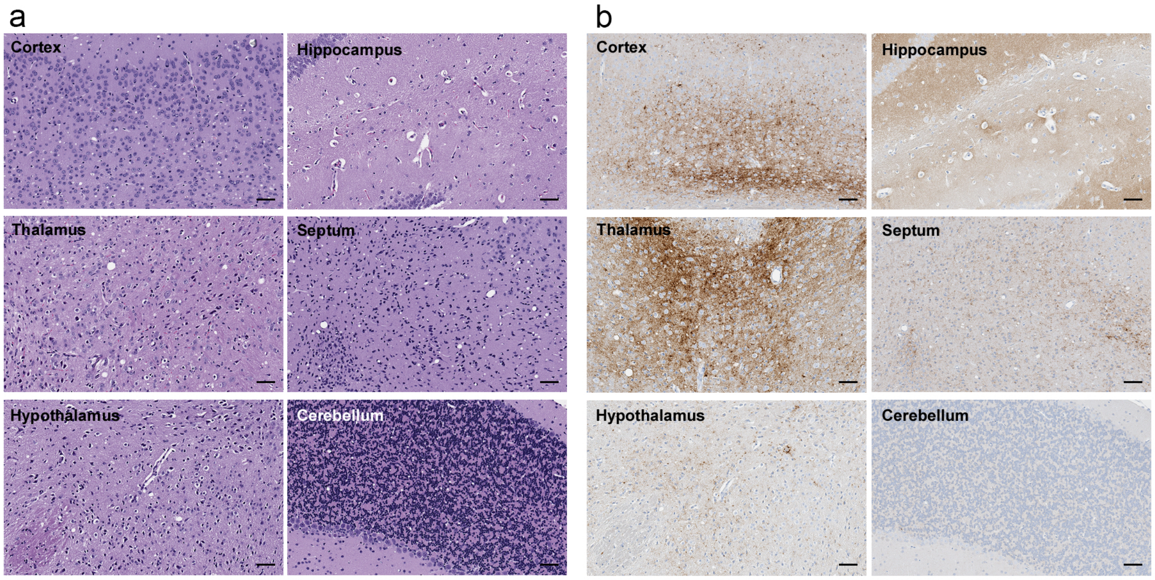

Supplementary Fig. 2 Spongiform change and PrP^{Sc} deposition in tgRM mice inoculated with MM1 sCJD a) H&E staining of tgRM mice inoculated with MM1 sCJD. The incubation time to disease was 204 dpi for all panels except for the hippocampus, thalamus, and hypothalamus which were from a mouse with an incubation time of 219 dpi. b) PrP^{Sc} deposition in tgRM mice inoculated with MM1 sCJD. The fields shown are matched to those in panel a. Sections were stained using the mouse monoclonal anti-PrP antibody 3F4 conjugated to biotin. For both panels, the brain region shown is indicated in the upper left-hand corner of each panel. For all panels, scale bar = 50 microns.

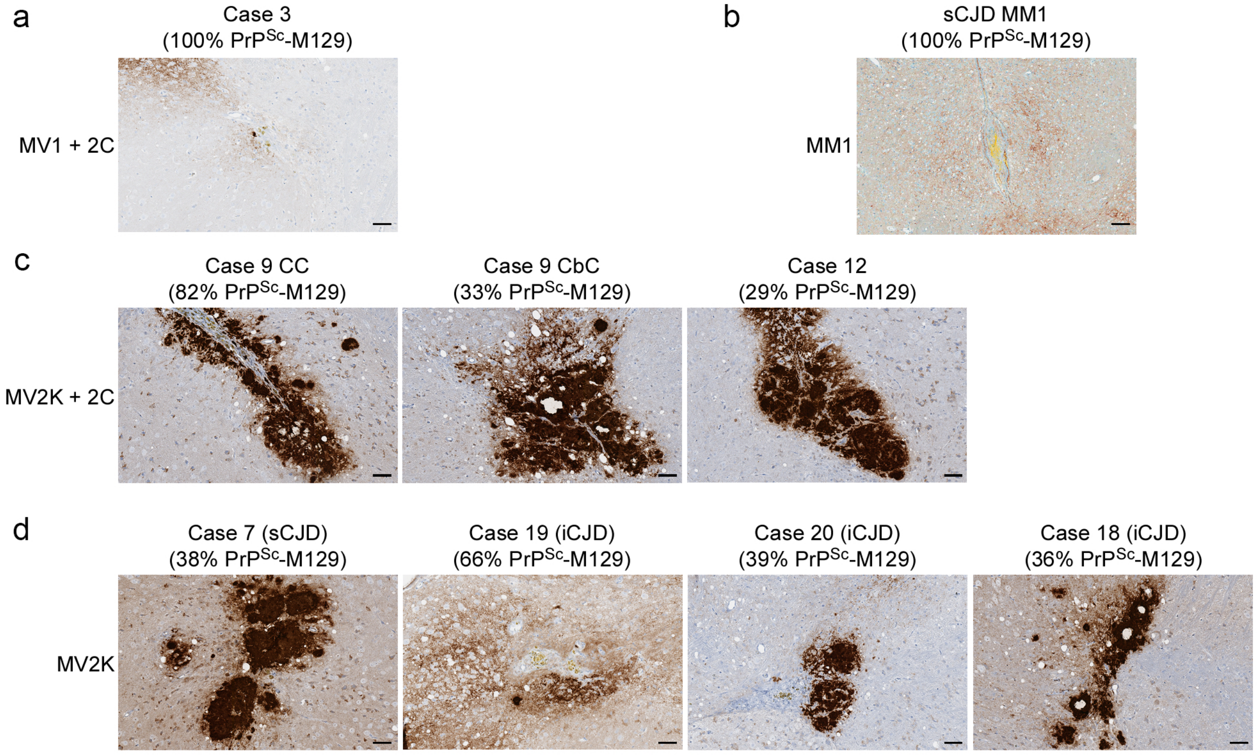

Supplementary Fig. 3 PrP^{Sc} positive amyloid plaques around the needle scar in transgenic mice inoculated with brain homogenate from MV heterozygous cases of CJD a) Tg66 mouse inoculated with MV1 + 2C case 3 (183 dpi). b) Tg66 mouse inoculated with MM1 sCJD (177 dpi). c) Tg66 mice inoculated with MV2K + 2C case 9 CC (502 dpi, left panel) and case 9 CbC (481 dpi, middle panel). TgRM mouse inoculated with case MV2K + 2C case 12 (471 dpi, right panel). d) Tg66 mice inoculated with MV2K sCJD case 7 (386 dpi, first panel) and MV2K iCJD cases 19 (185 dpi, second panel), 20 (458 dpi, third panel), and 18 (360 dpi, fourth panel). The mean percentage of PrP^{Sc}-M129 in each sample is given under the case number. All sections were stained using the mouse monoclonal anti-PrP antibody 3F4 conjugated to biotin. For all panels, only the area around the needle scar is shown and the scale bar = 50 microns.

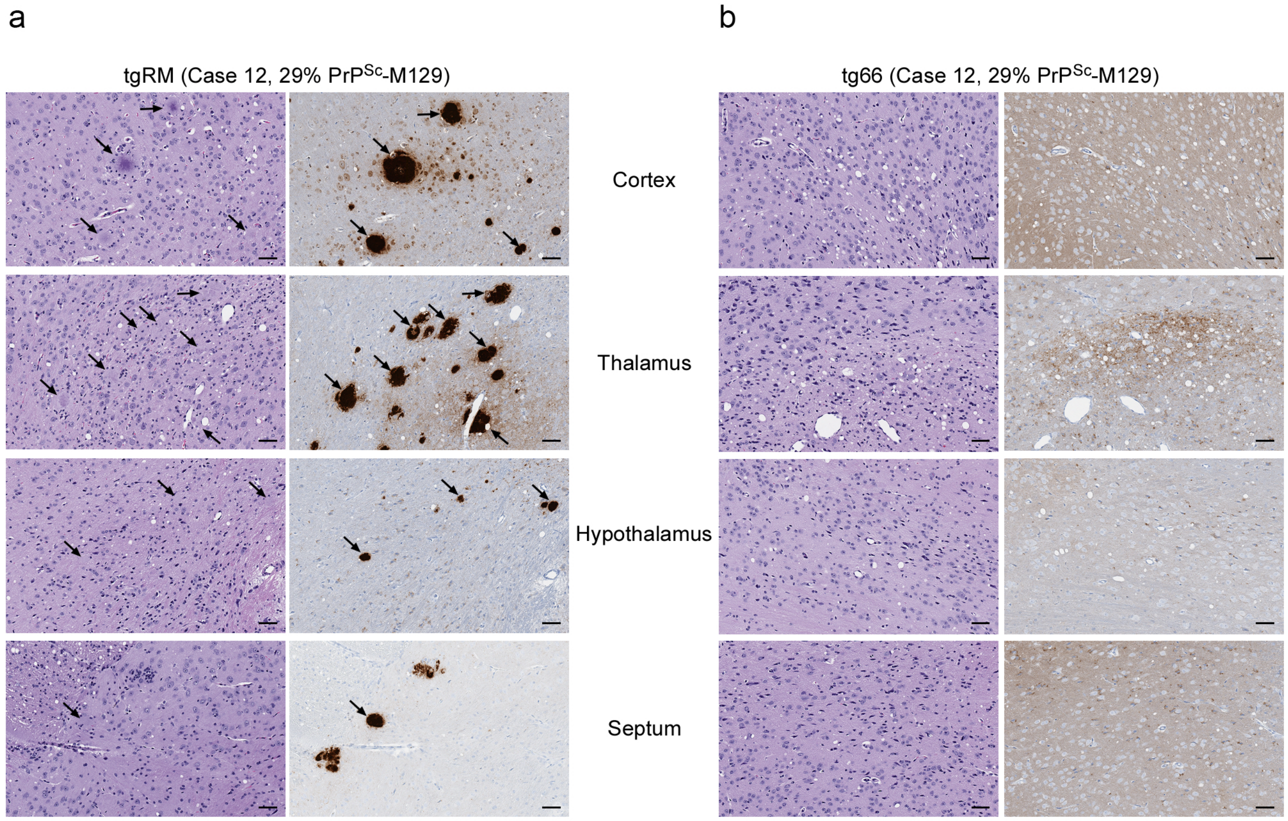

Supplementary Fig. 4 Spongiform change and PrP^{Sc} deposition differ in the brains of tg66 and tgRM mice inoculated with MV2K + 2C sCJD case 12 a) H&E (left column) and PrP^{Sc} staining (right column) of a tgRM mouse inoculated with MV2K + 2C sCJD case 12 (471 dpi). The black arrows indicate eosinophilic amyloid plaques that are also PrP^{Sc} positive. b) H&E (left column) and PrP^{Sc} staining (right column) of a tg66 mouse inoculated with MV2K + 2C sCJD case 12 (403 dpi). All PrP^{Sc} sections were stained using the mouse monoclonal anti-PrP antibody 3F4 conjugated to biotin. The mean percentage of PrP^{Sc}-M129 in each sample is given next to the case number. The brain regions shown are indicated in the middle of the figure. For all panels, scale bar = 50 microns.

Supplementary Table 1. Histopathological and neuropathological phenotypes of tg66 and tgRM mice inoculated with heterozygous cases of sCJD and iCJD with variable PrP^{Sc} allotypes.

		Neuropathological and Histopathological Features[a]	
**Case**	**Subtype**	**tg66**	**tgRM**
Control	MM1	-Spongiform change: mild to moderate in thalamus, hypothalamus, hippocampus, and septum with some microvacuoles in cortex and cerebellum.<br><br>-PrP^{Sc}: Widespread diffuse, punctate/synaptic.	-Spongiform change: mild but similar to tg66 in distribution.<br><br>-PrP^{Sc}: similar to tg66.
3	MV1 + 2C	- Spongiform change: mild to moderate in thalamus, hypothalamus, and cortex; cerebellum and hippocampus negative.<br><br>-PrP^{Sc}: Diffuse, punctate/synaptic, in same regions as above; cerebellum and hippocampus negative.	-Spongiform change: Similar to tg66 except mild to moderate with greater involvement of cortex.<br><br>-PrP^{Sc}: Distribution and deposition similar to tg66 except with stronger staining in the cortex.
4	MV1 + 2C	-Spongiform change: severe in cortex; moderate in thalamus, hippocampus, and granular layer of cerebellum.<br><br>-PrP^{Sc}: Punctate, synaptic, and dense perivacuolar deposits with occasional amyloid plaques and plaque-like deposits in thalamus, cortex, hypothalamus, hippocampus, and granular layer of cerebellum.	-Spongiform change: Distribution and severity similar to tg66 mice. One mouse was negative.<br><br>-PrP^{Sc}: Distribution and deposition similar to tg66 mice. One mouse was negative.
9 (CC)	MV2K + 2C	-Spongiform change: Mild to moderate in cortex, thalamus, and hypothalamus; moderate in septum. One mouse was negative.<br><br>-PrP^{Sc}: Dense perivacuolar and plaque-like deposits in cortex, septum, hypothalamus, and thalamus with dense amyloid plaques around needle scar. Some diffuse, punctate/synaptic deposits mainly in midbrain and granular layer of cerebellum. One mouse was negative.	-Spongiform change: Only 1/3 mice showed pathology. Mild, primarily in midbrain, septum, and thalamus.<br><br>-PrP^{Sc}: Only 1/3 mice showed pathology. Diffuse, punctate/synaptic mainly in midbrain and granular layer of cerebellum with plaques and plaque-like deposits in cortex, septum and thalamus.

		Neuropathological and Histopathological Features[a]	
**Case**	**Subtype**	**tg66**	**tgRM**
9 (CbC)	MV2K + 2C	-Spongiform change: Mild in cortex and moderate in thalamus and hypothalamus. Septum negative.<br><br>-PrP^{Sc}: Scattered unicentric and multicentric amyloid plaques with plaque-like and perivacuolar deposits; large plaques at needle scar; diffuse, in granular layer of cerebellum.	-Spongiform change: Mild with distribution similar to tg66 except that cortex is negative.<br><br>-PrP^{Sc}: Distribution similar to tg66 but with less intense staining and no cortical stain.
12	MV2K + 2C	-Spongiform change: Mild to moderate mainly in thalamus, hypothalamus, septum, hippocampus, cortex, granular layer of cerebellum. One mouse was negative.<br><br>-PrP^{Sc}: Diffuse, punctate/synaptic in cortex, thalamus, hypothalamus, granular layer of cerebellum. One mouse negative.	-Spongiform change: Only one of two mice had significant pathology. Mild mainly in thalamus, hypothalamus, septum, hippocampus, cortex; cerebellum negative.<br><br>-PrP^{Sc}: Scattered amyloid plaques and plaque-like deposits in cortex, septum, thalamus, hypothalamus; large, amyloid plaques at needle scar.
7	MV2K	-Spongiform change: Moderate mainly in thalamus, hypothalamus, corpus callosum, and hippocampus; mild in cortex and cerebellum.<br><br>-PrP^{Sc}: Amyloid plaques in most regions including thalamus, hypothalamus, cortex, cerebellum, and hippocampus with amyloid plaques at needle scar; occasional diffuse, punctate/synaptic, perivacuolar, and plaque-like deposition.	-Spongiform change: Similar distribution to tg66 but less severe.<br><br>-PrP^{Sc}: Similar distribution to tg66.
10	MV2K	-Spongiform change: as in case 7<br><br>-PrP^{Sc}: as in case 7	-Spongiform change: as in case 7<br><br>-PrP^{Sc}: as in case 7

		Neuropathological and Histopathological Features[a]	
**Case**	**Subtype**	**tg66**	**tgRM**
18 (iCJD)	MV2K	-Spongiform change: Mild to moderate in most brain regions; cerebellum negative.<br><br>-PrP^{Sc}: Primarily diffuse or punctate/synaptic mainly in cortex, thalamus, and hypothalamus, with scattered plaque-like deposits; amyloid plaques around needle scar.	-Spongiform change: Similar distribution to tg66 but less severe.<br><br>-PrP^{Sc}: Similar distribution to tg66; amyloid plaques around needle scar.
19 (iCJD)	MV2K	-Spongiform change: One sample available to analyze. Mild to moderate with distribution as in case 18.<br><br>-PrP^{Sc}: One sample available to analyze; distribution and deposition as in case 18.	-Spongiform change: Mild to moderate as in case 18.<br><br>-PrP^{Sc}: Distribution and deposition as in case 18; large plaques around needle scar.
20 (iCJD)	MV2K	-Spongiform change: Mild to moderate with distribution similar to cases 18 and 19. One mouse negative.<br><br>-PrP^{Sc}: Diffuse deposition less prominent than in cases 18 and 19 with increased abundance of plaque-like deposits; amyloid plaques around needle scar. One mouse negative.	- Spongiform change: Only 1/3 mice showed pathology similar to case 18.<br><br>-PrP^{Sc}: Only 1/3 mice positive with distribution and deposition similar to case 18.
11	MV2C	-Spongiform change: Mild spongiform change primarily in cortex and hippocampus.<br><br>-PrP^{Sc}: Punctate/synaptic and dense deposits, some perivacuolar, primarily in cortex, septum, corpus collosum, and hippocampus.	-Spongiform change: Not above age-related background levels.<br><br>-PrP^{Sc}: Dense deposits, that are occasionally perivacuolar, primarily in cortex and thalamus.

a = based on analysis of 3-4 mice except where noted.
